# Case Report: Biallelic variants in *MRPS36*, encoding a component of the 2-oxoglutarate dehydrogenase complex, cause leigh syndrome

**DOI:** 10.3389/fped.2025.1608840

**Published:** 2025-09-12

**Authors:** Huafang Jiang, Chaolong Xu, Zhimei Liu, Ruoyu Duan, Xingfeng Yao, Xiaona Fu, Jiatong Xu, Xuejing Kang, Tenghui Yu, Yuanyuan Wang, Fang Fang

**Affiliations:** ^1^ Department of Pediatrics, Weifang Maternal and Child Health Hospital, Peking University Health Science Center-Weifang Joint Research Center for Maternal and Child Health, Weifang, China; ^2^ Department of Neurology, Beijing Children’s Hospital, Capital Medical University, National Center for Children’s Health, Beijing, China; ^3^ Department of Pathology, Beijing Children’s Hospital, Capital Medical University, National Center for Children’s Health, Beijing, China

**Keywords:** *MRPS36* gene, 2-oxoglutarate dehydrogenase complex, OGDHC, Leigh syndrome, movement disorder, choreic movement, case report

## Abstract

**Background:**

The *MRPS36* gene encodes the E4 subunit of the 2-oxoglutarate dehydrogenase complex (OGDHC), a critical enzyme in the tricarboxylic acid cycle. OGDHC deficiency can lead to metabolic disorders with a clinical spectrum ranging from fatal neonatal lactic acidosis to variable degrees of global developmental delay and movement disorders. To date, a homozygous *MRPS36* variant has been reported as a novel cause of Leigh syndrome in only two siblings, who presented with developmental delay, movement disorders, bilateral striatal necrosis, and reduced OGDHC activity.

**Case presentation:**

We report a third case of Leigh syndrome associated with *MRPS36* variants in a 2-year-old boy. The patient exhibited with global developmental delay, dystonia, early-onset chorea, and elevated serum lactate levels. Follow-up brain magnetic resonance imaging at 2 years revealed progressive degenerative lesions in the bilateral basal ganglia. Muscle biopsy showed abnormal mitochondrial accumulation beneath the sarcolemma, and the oxygen consumption rate was reduced in skin fibroblasts. Whole-exome sequencing identified two novel compound heterozygous *MRPS36* variants: c.42+1G>A (p.?) and c.296G>C (p.Arg99Pro).

**Conclusion:**

This case supports *MRPS36* as a novel pathogenic cause of Leigh syndrome, further expanding the genetic spectrum of the disorder. Key clinical features include developmental delay, involuntary movement disorders, progressive basal ganglia atrophy, and a slowly progressive disease course.

## 1 Introduction

Leigh syndrome (LS; OMIM#25600), also referred to as subacute necrotizing encephalomyelopathy, is a clinically and genetically heterogeneous neurodegenerative disorder (1). As the most common pediatric presentation of mitochondrial disease, LS has been linked to pathogenic variants in over 100 genes involved in pyruvate metabolism, the tricarboxylic acid (TCA) cycle, and oxidative phosphorylation (2, 3). The 2-oxoglutarate dehydrogenase complex (OGDHC) catalyzes the conversion of *α*-ketoglutarate to succinyl-CoA, generating NADH and releasing CO_2_ within the TCA cycle (4). OGDHC is a multisubunit enzyme complex composed of 2-oxoglutarate dehydrogenase (OGDH)/E1(OMIM*613022), dihydrolipoamide succinyltransferase (DLST)/E2(OMIM*126063), and dihydrolipoamide dehydrogenase (DLD)/E3 (OMIM*238331) (5). Mitochondrial ribosomal protein S36 (MRPS36)/E4 (OMIM*611996) was recently identified as an adaptor protein that stabilizes the association of E3 with the E1-E2 core (5, 6). In 2024, Galosi et al. reported two siblings with a homozygous *MRPS36* variant, establishing this gene as a novel cause of LS (7). Both individuals exhibited severe developmental delays, generalized dystonia, prominent movement disorders, bilateral striatal necrosis, and markedly reduced OGDHC activity.

Here, we describe a third individual with LS associated with compound heterozygous variants in *MRPS36*, presenting with global developmental delay and early-onset choreic movements. Over a two-year follow-up period, the disease course was slowly progressive, with serial brain magnetic resonance imaging (MRI) revealing progressive basal ganglia degeneration. The clinical and radiological findings were consistent with previously reported cases, further supporting the role of *MRPS36* as a gene implicated in LS pathogenesis.

## 2 Methodology

### 2.1 Patient

The patient was initially admitted to Weifang Maternal and Child Health Hospital and subsequently followed up at Beijing Children's Hospital, Capital Medical University. This study was approved by the Ethics Committee of Beijing Children's Hospital. Informed consent was obtained from the patient's parents. All procedures were conducted in accordance with the principles outlined in the Declaration of Helsinki. This case report was prepared in compliance with the CARE (CAse REport) guidelines.

### 2.2 Genetic analysis

Total DNA was extracted from peripheral blood samples obtained from the patient and family members. Whole exome sequencing (WES) was performed by Chigene Genomics Corporation using the IDT xGen Exome Research Panel v2.0 on an Illumina HiSeq X Ten sequencer. Sanger sequencing was used to confirm the presence of identified variants.

### 2.3 Muscle biopsy

A muscle biopsy was obtained from the left biceps brachii and examined using light and electron microscopy. Samples were pre-cooled in isopentane and cryofixed in liquid nitrogen. Tissue sections were processed for routine enzyme histochemical staining and mitochondrial respiratory chain complex activity assays, including complexes I, II, II + III, III, IV, and the mitochondrial marker enzyme citrate synthase.

### 2.4 Mitochondrial respiration

Skin fibroblasts were obtained from a skin biopsy and cultured in Dulbecco's Modified Eagle Medium (DMEM, Thermo Fisher Scientific) supplemented with 10% fetal bovine serum and 1% penicillin–streptomycin. The mitochondrial oxygen consumption rate (OCR) was measured using an XF96 Extracellular Flux Analyzer (Agilent Technologies, USA). Basal respiration, adenosine triphosphate (ATP) production, and maximal respiration were calculated from the OCR data.

## 3 Case presentation

### 3.1 Clinical features

The proband was born full-term to healthy, non-consanguineous Chinese parents. The family history included a prior pregnancy that ended in an induced abortion and a healthy 10-year-old sister. During the neonatal period, the proband exhibited irritability and excessive movements. At 4 months of age, he presented with poor head control. At that time, developmental milestones were limited to visual tracking, auditory response, and unsteady head support. Rehabilitation therapy was initiated. At 6 months, he had mild improvement in motor function, including the ability to roll over. By 1 year of age, the patient developed involuntary movements such as grimacing, tongue protrusion, brow furrowing, and limb chorea. Neurological examination revealed choreiform movements, dystonia, and brisk tendon reflexes. At 2 years of age, the clinical status remained stable, with persistent chorea and limb spasticity, both of which worsened with emotional stress or infections. The patient was able to sit with assistance and comprehend simple sentences but had not developed spoken language. Marked global motor developmental delay was observed, with relatively preserved of language comprehension and social interaction.

Biochemical testing revealed a mildly elevated creatine kinase level [392 IU/L; reference range (r.r.): 38–174 IU/L]. Serum lactate levels remained consistently elevated (2.6–3.4 mmol/L; r.r. <2.2 mmol/L). Urine organic acid and plasma amino acid analysis showed mildly increased levels of medium- and long-chain fatty acids. Electrocardiography revealed a prolonged PR interval, while echocardiography findings were unremarkable. Electroencephalograms performed at 4 months and at 2 years 3 months were normal.

Treatment included carnitine (1 g/day), niacinamide (50 mg/day), and coenzyme Q10 (100 mg/day) to support mitochondrial function. Tiapride was administered to alleviate movement disorders; however, no clinical improvement were noted.

### 3.2 Imaging analysis

**Figure 1:**
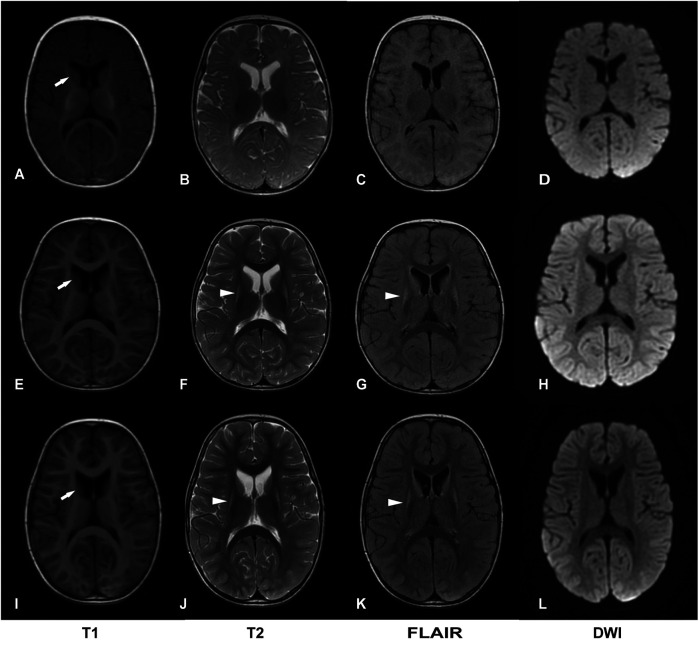
Two-year follow-up of brain magnetic resonance imaging (MRI). **(A–D)** Normal brain images at 4 months of age. **(E–H)** Bilateral symmetric lesions in the basal ganglia, without restricted diffusion at 17 months of age. **(I–L)** Symmetric putamen lesions with atrophy of the head of the caudate nucleus at 2 years and 3 months of age. DWI, diffusion-weighted imaging. Basal ganglia lesions are indicated by arrowheads; atrophy of the head of the caudate nucleus is indicated by white arrows.

The initial brain MRI at 4 months of age was unremarkable (Figures 1A–D). A follow-up MRI at 17 months revealed bilateral, symmetric T2-weighted hyperintensities in the basal ganglia without restricted diffusion (Figures 1E–H). At 2 years and 3 months of age, brain MRI showed symmetric lesions in the putamen and atrophy of the caudate head (Figures 1I–L).

### 3.3 Histopathological analysis

**Figure 2:**
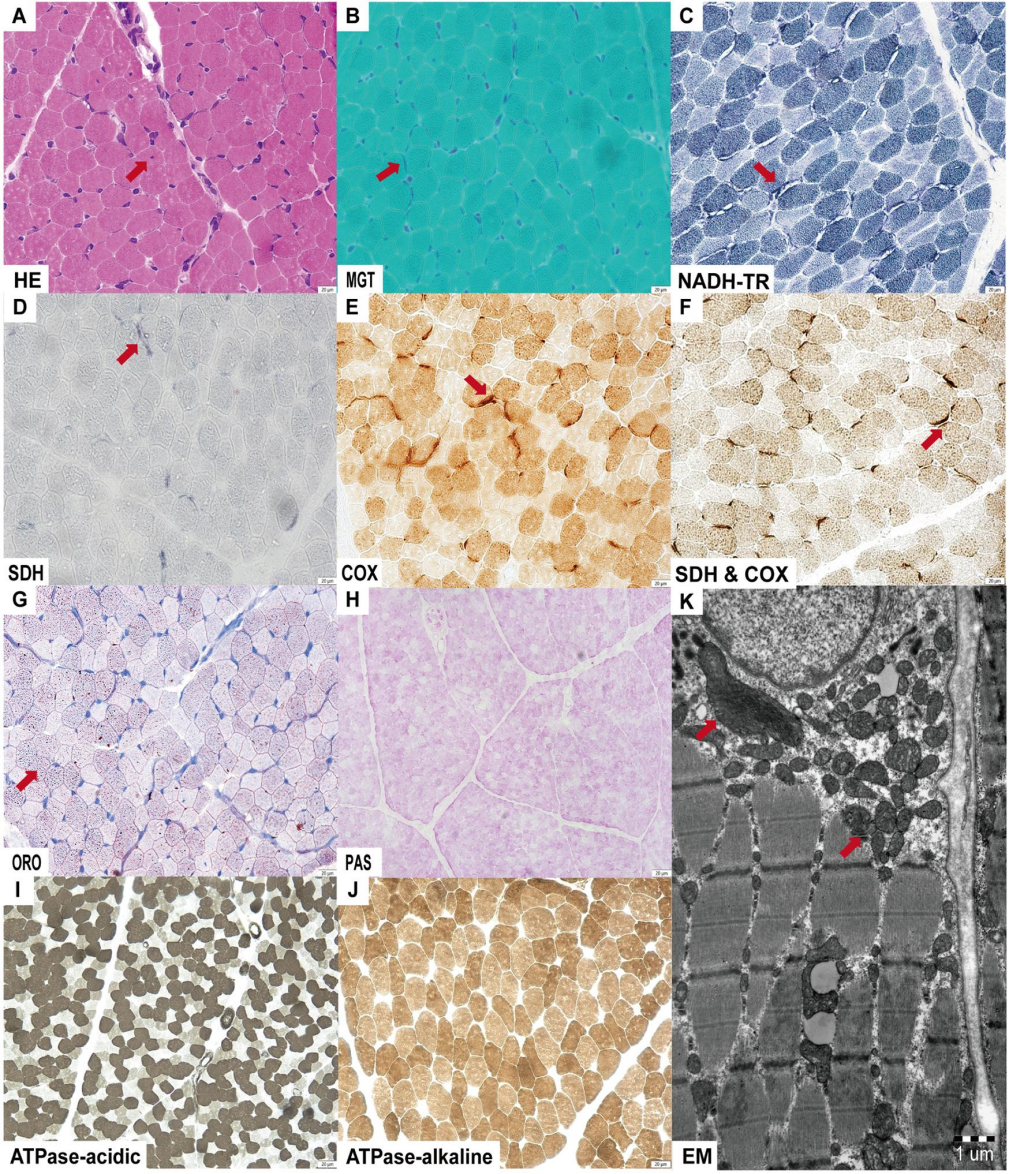
Muscle histopathological alterations were observed by light and electron microscopy. **(A)** Nuclear invagination on hematoxylin and eosin (HE) staining; **(B–F)** Submembranous deep staining observed for MGT, SDH, COX and SDH & COX staining; NADH-TR staining revealed submembranous gaps with deep staining at the edges; **(G)** Mild lipid droplet accumulation on ORO staining; **(H–J)** No abnormalities detected on Periodic-Acid Schiff (PAS) and ATPase staining. **(K)** Electron microscopy (EM) shows accumulation of mitochondria with variable sizes and shapes.

Light microscopy of the muscle biopsy revealed mild lipid droplet accumulation and deep submembranous staining for MGT, SDH, and COX in select fibers. NADH-TR staining showed submembranous gaps in certain fibers, with intendified staining at the gap margins. Electron microscopy demonstrated an increased number of mitochondria with variable size and morphology between the myofilaments and beneath the sarcolemma (Figure 2).

### 3.4 Mitochondrial function analysis

**Figure 3:**
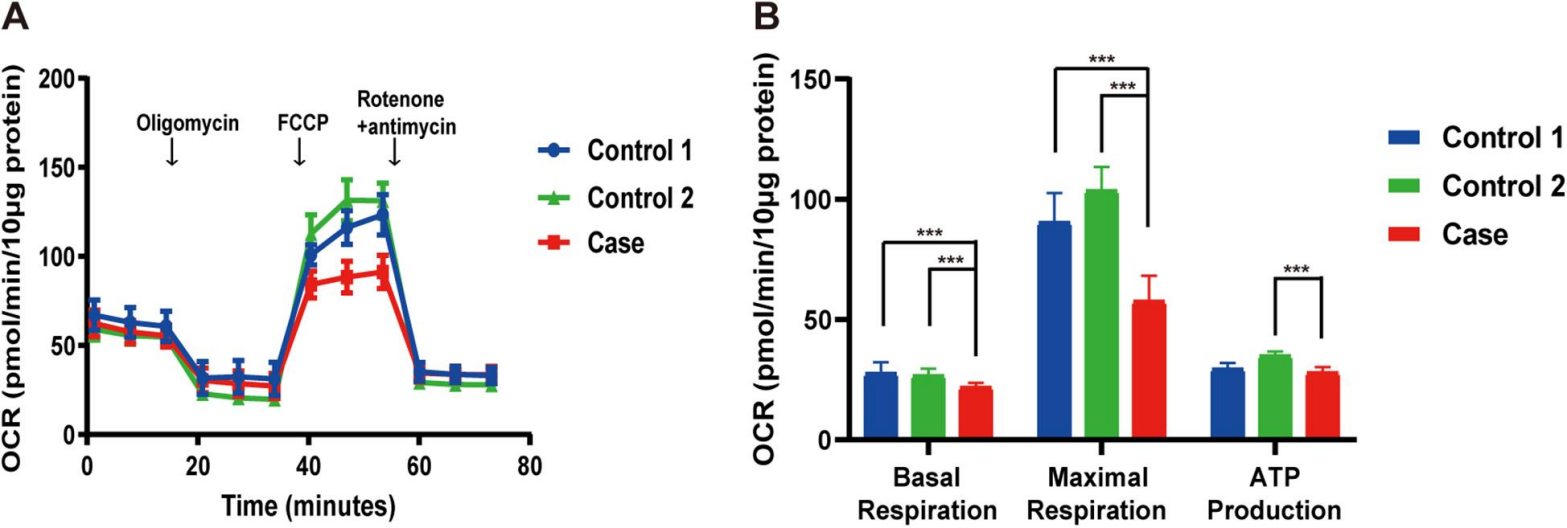
Reduced OCR in the patient's fibroblasts. **(A)** Overview of the OCR from the patient compared to controls. **(B)** Graphs of basal respiration, maximal respiration, and ATP production calculated from the OCR. Data are indicated as mean ± SD. Significance calculated by a one-way ANOVA test. FCCP, carbonyl cyanide phenylhydrazone; OCR, oxygen consumption rate. ***P < 0.001.

Mitochondrial respiratory chain complex activities in the muscle were within normal ranges. However, fibroblast-based analysis showed reduced basal respiration, maximal respiration, and ATP production, as measured by OCR, indicating impaired mitochondrial respiration (Figure 3).

### 3.5 Genetic analysis

**Figure 4:**
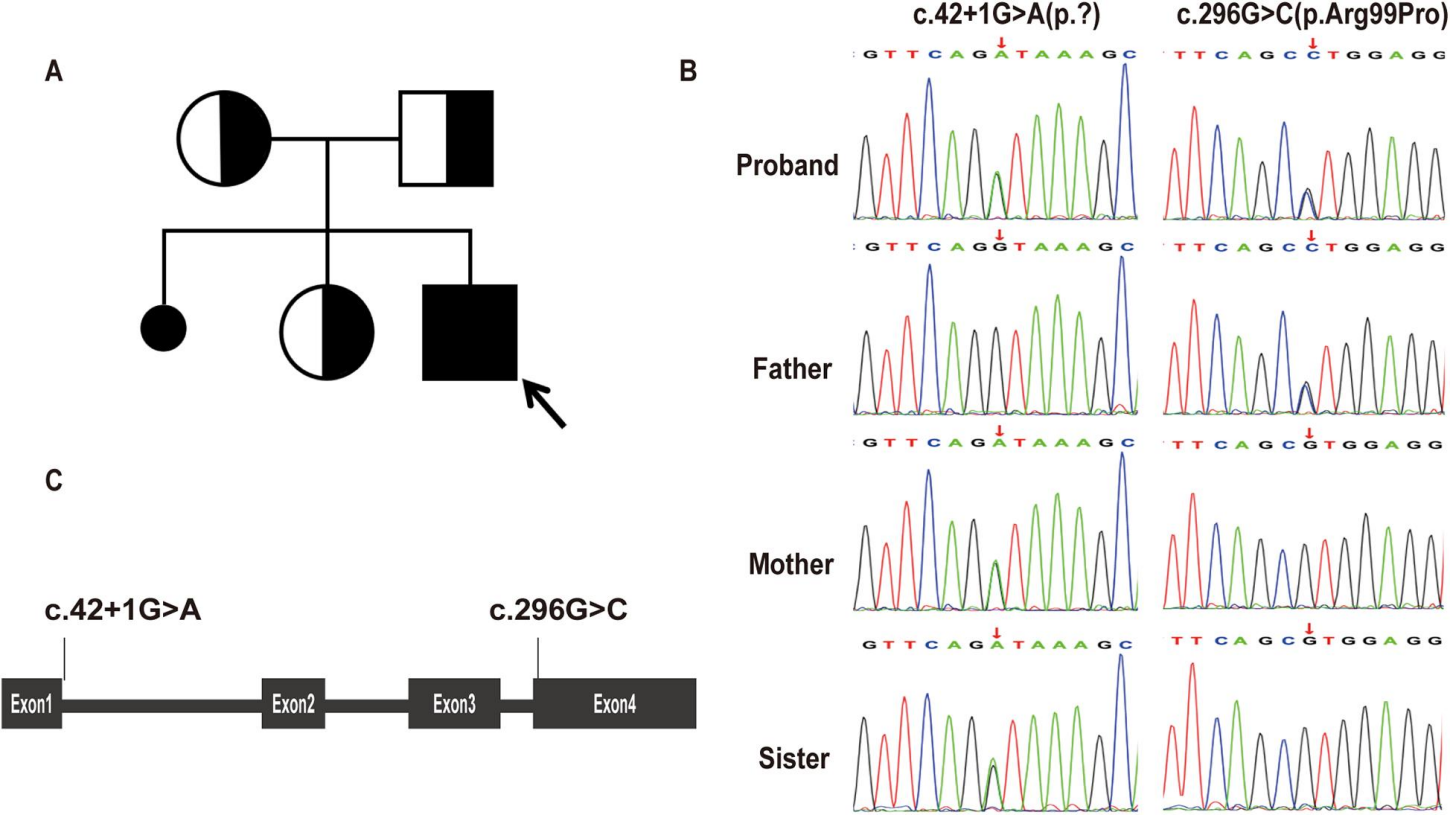
Genetic findings of the proband. **(A)** Pedigree chart. The proband was indicated by the arrow. **(B)** Sanger sequencing of *MRPS36* variants in the proband and his family members. The patient, his mother, and older sister carried the c.42+1G>A(p.?), while the patient and his father carried the c.296G>C(p.Arg99Pro). **(C)** Schematic diagram of *MRPS36* organization illustrates the locations of the variants identified in our report.

A clinical diagnosis of LS was made based on developmental delay, persistent lactic acidemia, bilateral symmetric basal ganglia lesions on MRI, and reduced mitochondrial OCR. Genetic analysis indetified compound heterozygous variants in the MRPS36 gene (NM_033281.5): c.42+1G>A (p.?) and c.296G>C (p.Arg99Pro). Sanger sequencing confirmed the variants were inherited from each parent. The proband's healthy sister carried the c.42+1G>A (p.?) variant (Figure 4). The clinical phenotype was consistent with previously reported cases involving *MRPS36* mutation (7). The c.42+1G>A variant affacts the canonical +1 splice site and is absent from the 1,000 Genomes Project and gnomAD databases (8, 9). Base on American College of Medical Genomics (ACMG) criteria, this variant was classified as pathogenic (PVS1, PM2, PP4) (6, 10, 11). The c.296G>C variant, also absent from population databases, was predicted to be damaging by multiple in silico prediction tools (SIFT: Damaging; PolyPhen-2: Probably damaging; MutationTaster: Disease causing) (8, 9, 12–14). According to ACMG guidelines, it was classified as likely pathogenic (PM2, PM3, PP3, PP4) (11). Both variants have been submitted to ClinVar.

## 4 Discussion

The TCA cycle is a crucial metabolic pathway that integrates glucose, lipid, and protein metabolism and is critical for aerobic respiration (15). Deficiencies in TCA cycle enzymes can result in severe early-onset neurological disorders with underlying metabolic dysfunction. One each enzyme complex, the OGDHC, catalyzes the rate-limiting conversion of 2-oxoglutarate to succinyl-CoA (16). OGDHC is a large multimeric enzyme complex compose of three catalytically active subunits (OGDH/E1, DLST/E2, and DLD/E3) and a recruiting subunit, MRPS36.

To date, 18 patients have been reported with OGDHC deficiency. Seven of these cases presented with phenotypes ranging from fatal neonatal lactic acidosis to global developmental delay, progressive movement disorders, and epilepsy, but lacked confirmed molecular diagnoses (7, 17–24). In 2020, Yap et al. identified *OGDH*/E1 as a pathogenic gene associated with OGDHC deficiency (23). Subsequently, biallelic mutations in *OGDH* were described in six patients, all of whom exhibited global developmental delay, hypotonia, dystonia, and elevated serum lactate levels—findings consistent with OGDHC dysfunction (24). *DLST*/E2, a gene known to act as a tumor suppressor, has not yet been implicated in primary mitochondrial disorders (25). The DLD gene encodes the E3 subunit, which is a shared across multiple dehydrogenase complexes, including OGDHC, the pyruvate dehydrogenase complex, and the branched-chain *α*-ketoacid dehydrogenase complexes (26). As a result, *DLD* deficiency typically causes severe neonatal-onset metabolic decompensation, with features such as lethargy, hypotonia, seizures, developmental delay, and ataxia, and frequently leads to early death (27). In contrast, late-onset *DLD* deficiency can manifest as hepatic dysfunction and myopathy (28).

In 2024, *MRPS36*, encoding the E4 subunit of OGDHC, was reported to harbor a homozygous truncating variant in two sibling with LS (7). These siblings displayed a spectrum of LS characterized by severe developmental delay, preserved social interaction and language comprehension, generalized dystonia, and hyperkinetic episodes precipitated by emotional or physical stress. Brain MRI revealed symmetric putaminal lesions consistent with striatal necrosis and diffuse cerebral atrophy. Biochemical investigations showed mildly elevated plasma glutamate and glutamine, suggestive of impaired OGDHC activity. Here, we report the third case of LS associated with biallelic *MRPS36* variants, furthering supporting its role as a diaease-causing gene. Our patient exhibited developmental delay, dystonia, and early-onset involuntary movements. Serial MRI over a two-year period showed disease progression—from a normal scan at 4 months of age to bilateral putamenial lesions and atrophy of the caudate head by 2 years. Muscle histopathology was unremarkable for classical mitochondrial features, such as ragged red fibers or COX-negative fibers. However, special stains (MGT, SDH, COX and NADH-TR) and ultrastructural studies demonstrated mitochondrial accumulation around myofibers, supporting underlying mitochondrial dysfunction. Furthermore, respiratory chain complex activity in muscle tissue remained normal, suggesting that *MRPS36* deficiency may not impair the mitochondrial respiratory chain directly. The clinical features of our patient were consistent with those of the 209 individuals with LS reported by our group in 2022, reinforcing *MRPS36* as a novel gene associated with LS (3). Unlike the previously reported siblings, our patient exhibited a structurally normal heart and no abnormalities in glutamine metabolism. Althouge the clincial course over two years was relatively stable, LS is neurodegenerative and longer-term follow-up is required to understand the trajectory of *MRPS36*-related disease. A limitation of this study is the absence of functional assays directly assessing OGDHC enzymatic activity or *MRPS36* transcript analysis.

Computational modeling by Zhang et al. identified MRPS36 as a eukaryote-specific subunit that anchors the E3 subunit to the E1-E2 core of OGDHC via its N- and C-terminal domains (4). Heublein et al. Further demonstrated that *MRPS36* deficiency led to reduced OGDH activity in mitochondrial lysates without destabilizing the catalytic subunits (6). As a binding protein, MRPS36 loss may result in partial OGDHC dysfunction, which could explain the milder and more stable phenotype observed in our patient compared with more severe neonatal-onset OGDHC deficiencies (12). Our patient also exhibited mild lipid droplet accumulation and increased urinary medium- and long-chain fatty acids, suggesting disturbances in fatty acid metabolism—possibly secondary to *α*-ketoglutarate accumulation. These findings argue against the use of a ketogenic diet and support the exploration of succinyl-CoA supplementation as a potential therapeutic approach. However, given the rarity of *MRPS36* deficiendy and the limited functional data available, further studies are essential to confirm the role of *MRPS36* in mitochondrial dysfunction.

## 5 Conclusions

This report describes a case of LS involving novel heterozygous variants in *MRPS36*. The patient exhibited developmental delay, dystonia, early-onset choreic movements, and putaminal lesions, along with atrophy of the caudate nucleus head on MRI. This case represents the second instance of *MRPS36* variants associated with LS and provides further support for *MRPS36* as a novel pathogenic cause to LS.

## Data Availability

The original contributions presented in the study are included in the article/Supplementary Material, further inquiries can be directed to the corresponding authors.

## References

[1] Rahman S Thorburn D Ball M. Nuclear gene-encoded leigh syndrome spectrum overview. In: Adam MP Feldman J Mirzaa GM Pagon RA Wallace SE Amemiya A, editors. GeneReviews®. Seattle (WA): University of Washington (1993–2025). 20301352

[2] Schubert Baldo M Vilarinho L. Molecular basis of leigh syndrome: a current look. Orphanet J Rare Dis. (2020) 15:31. 10.1186/s13023-020-1297-9 31996241 PMC6990539

[3] Stenton SL Zou Y Cheng H Liu Z Wang J Shen D Leigh syndrome: a study of 209 patients at the Beijing children’s hospital. Ann Neurol. (2022) 91:466–82. 10.1002/ana.26313 35094435

[4] Zhang Y Chen M Chen X Zhang M Yin J Yang Z Molecular architecture of the mammalian 2-oxoglutarate dehydrogenase complex. Nat Commun. (2024) 15:8407. 10.1038/s41467-024-52792-7 39333186 PMC11436768

[5] Hevler JF Albanese P Cabrera-Orefice A Potter A Jankevics A Misic J MRPS36 provides a structural link in the eukaryotic 2-oxoglutarate dehydrogenase complex. Open Biol. (2023) 13:220363. 10.1098/rsob.220363 36854377 PMC9974300

[6] Heublein M Burguillos MA Vogtle FN Teixeira PF Imhof A Meisinger C The novel component Kgd4 recruits the E3 subunit to the mitochondrial alpha-ketoglutarate dehydrogenase. Mol Biol Cell. (2014) 25:3342–9. 10.1091/mbc.e14-07-1178 25165143 PMC4214781

[7] Galosi S Mancini C Commone A Calligari P Caputo V Nardecchia F Biallelic variants of MRPS36 cause a new form of leigh syndrome. Mov Disord. (2024) 39:1225–31. 10.1002/mds.29795 38685873

[8] 1000 Genomes Project Consortium, Abecasis GR Auton A Brooks LD DePristo MA Durbin RM An integrated map of genetic variation from 1,092 human genomes. Nature. (2012) 491:56–65. 10.1038/nature11632 23128226 PMC3498066

[9] Karczewski KJ Francioli LC Tiao G Cummings BB Alföldi J Wang Q The mutational constraint spectrum quantified from variation in 141,456 humans. Nature. (2020) 581:434–43. 10.1038/s41586-020-2308-7 32461654 PMC7334197

[10] Walker LC Hoya M Wiggins GAR Vincent LA Parsons LM T M Using the ACMG/AMP framework to capture evidence related to predicted and observed impact on splicing: recommendations from the ClinGen SVI splicing subgroup. Am J Hum Genet. (2023) 110:1046–67. 10.1016/j.ajhg.2023.06.002 37352859 PMC10357475

[11] Richards S Aziz N Bale S Bick D Das S Gastier-Foster J Standards and guidelines for the interpretation of sequence variants: a joint consensus recommendation of the American college of medical genetics and genomics and the association for molecular pathology. Genet Med. (2015) 17:405–24. 10.1038/gim.2015.30 25741868 PMC4544753

[12] Ng PC Henikoff S. SIFT: predicting amino acid changes that affect protein function. Nucleic Acids Res. (2003) 31:3812–4. 10.1093/nar/gkg509 12824425 PMC168916

[13] Adzhubei I Jordan DM Sunyaev SR. Predicting functional effect of human missense mutations using PolyPhen-2. Curr Protoc Hum Genet. (2013): Chapter 7:Unit7.20. 10.1002/0471142905.hg0720s76 23315928 PMC4480630

[14] Schwarz JM Rödelsperger C Schuelke M Seelow D. Mutationtaster evaluates disease-causing potential of sequence alterations. Nat Methods. (2010) 7:575–6. 10.1038/nmeth0810-575 20676075

[15] Arnold PK Finley LWS. Regulation and function of the mammalian tricarboxylic acid cycle. J Biol Chem. (2023) 299:102838. 10.1016/j.jbc.2022.102838 36581208 PMC9871338

[16] Szabo E Nagy B Czajlik A Komlodi T Ozohanics O Tretter L Mitochondrial alpha-keto acid dehydrogenase complexes: recent developments on structure and function in health and disease. Subcell Biochem. (2024) 104:295–381. 10.1007/978-3-031-58843-3_13 38963492

[17] Chalmers RA Purkiss P Watts RW Lawson AM. Screening for organic acidurias and amino acidopathies in newborns and children. J Inherit Metab Dis. (1980) 3:27–43. 10.1007/BF02312520 6777599

[18] Kohlschutter A Behbehani A Langenbeck U Albani M Heidemann P Hoffmann G A familial progressive neurodegenerative disease with 2-oxoglutaric aciduria. Eur J Pediatr. (1982) 138:32–7. 10.1007/BF00442325 7075624

[19] Bonnefont JP Chretien D Rustin P Robinson B Vassault A Aupetit J Alpha-ketoglutarate dehydrogenase deficiency presenting as congenital lactic acidosis. J Pediatr. (1992) 121:255–8. 10.1016/S0022-3476(05)81199-0 1640293

[20] Guffon N Lopez-Mediavilla C Dumoulin R Mousson B Godinot C Carrier H 2-Ketoglutarate dehydrogenase deficiency, a rare cause of primary hyperlactataemia: report of a new case. J Inherit Metab Dis. (1993) 16:821–30. 10.1007/BF00714273 8295396

[21] al Aqeel A Rashed M Ozand PT Gascon GG Rahbeeni Z al Garawi S A new patient with alpha-ketoglutaric aciduria and progressive extrapyramidal tract disease. Brain Dev. (1994) 16(Suppl):33–7. 10.1016/0387-7604(94)90094-9 7726379

[22] Dunckelmann RJ Ebinger F Schulze A Wanders RJ Rating D Mayatepek E. 2-ketoglutarate dehydrogenase deficiency with intermittent 2-ketoglutaric aciduria. Neuropediatrics. (2000) 31:35–8. 10.1055/s-2000-15295 10774994

[23] Yap ZY Strucinska K Matsuzaki S Lee S Si Y Humphries K A biallelic pathogenic variant in the OGDH gene results in a neurological disorder with features of a mitochondrial disease. J Inherit Metab Dis. (2021) 44:388–400. 10.1002/jimd.12248 32383294 PMC7647956

[24] Whittle EF Chilian M Karimiani EG Progri H Buhas D Kose M Biallelic variants in OGDH encoding oxoglutarate dehydrogenase lead to a neurodevelopmental disorder characterized by global developmental delay, movement disorder, and metabolic abnormalities. Genet Med. (2023) 25:100332. 10.1016/j.gim.2022.11.001 36520152 PMC9905285

[25] Mellid S Garcia F Leandro-Garcia LJ Diaz-Talavera A Martinez-Montes AM Gil E DLST mutations in pheochromocytoma and paraganglioma cause proteome hyposuccinylation and metabolic remodeling. Cancer Commun (Lond). (2023) 43:838–43. 10.1002/cac2.12427 37139660 PMC10354410

[26] Pode-Shakked B Landau YE Shaul Lotan N Manor J Haham N Kristal E The natural history of dihydrolipoamide dehydrogenase deficiency in Israel. J Inherit Metab Dis. (2024) 47:895–902. 10.1002/jimd.12778 39040027

[27] Wongkittichote P Cuddapah SR Master SR Grange DK Dietzen D Roper SM Biochemical characterization of patients with dihydrolipoamide dehydrogenase deficiency. JIMD Rep. (2023) 64:367–74. 10.1002/jmd2.12382 37701333 PMC10494496

[28] Mihaljevic M Petkovic Ramadza D Zigman T Rako I Galic S Matic T Dihydrolipoamide dehydrogenase deficiency in five siblings with variable phenotypes, including fulminant fatal liver failure despite good engraftment of transplanted liver. JIMD Rep. (2024) 65:323–9. 10.1002/jmd2.12444 39544687 PMC11558464

